# The TET protein family interactor PROSER1 sustains hematopoietic stem cell function

**DOI:** 10.1182/bloodadvances.2024015683

**Published:** 2025-06-26

**Authors:** Elena V. Knatko, Anna Fleming, Xiang Li, Phoebe Crawley, Ieva Budriunaite, Kasper D. Rasmussen

**Affiliations:** Division of Molecular, Cell, and Developmental Biology, University of Dundee, Dundee, United Kingdom

## Abstract

Ten-eleven translocation (TET) enzymes are epigenetic regulators important for the prevention of hematological malignancies. Proline and serine rich 1 (PROSER1), a known TET protein interactor, has a critical role in modulating TET-mediated DNA demethylation during development. However, the potential involvement of PROSER1 in the regulation of hematopoiesis and leukemogenesis remains unknown. Here, we demonstrate that the leukemia-suppressive functions of TET2 are preserved in the absence of PROSER1. Nonetheless, we find that loss of PROSER1 partially recapitulates the aberrant enhancer DNA methylation phenotype observed upon TET2 knockout, suggesting that PROSER1 and TET2 play both cooperative and distinct roles in the regulation of DNA methylation in hematopoiesis. Importantly, using serial hematopoietic stem cell (HSC) transplantation assays, we find progressive exhaustion of HSC activity and reduction in hematopoietic lineage output upon loss of PROSER1. Our findings imply that, beyond the established role of TET2 loss-of-function mutations in promoting HSC expansion and leukemic transformation, accurate TET activity, regulated by PROSER1, is equally important to prevent HSC exhaustion and sustain normal hematopoiesis.

## Key Points

• PROSER1 deficiency results in dysregulation of DNA methylation patterns in hematopoietic progenitor cells.
• Loss of PROSER1 impairs HSC function without compromising TET-mediated leukemia suppression.

## Visual Abstract

**Figure:**
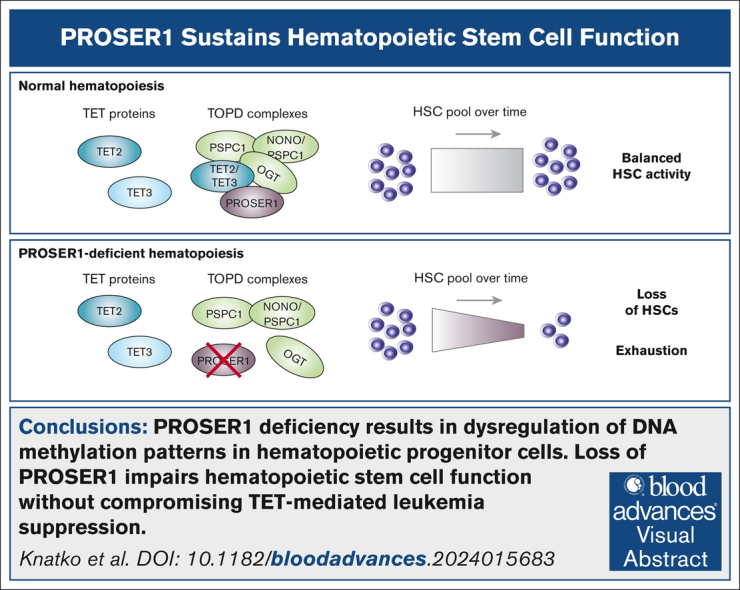

## Introduction

The ten-eleven translocation (TET) protein family consists of 3 members: TET1, TET2, and TET3.^1^ These closely related proteins contain a conserved catalytic domain that can iteratively oxidize 5-methylcytosine, promoting DNA demethylation and antagonizing the activity of DNA methyltransferase enzymes.^2^^,^^3^ Although all 3 TET enzymes have been shown to have a role in hematopoiesis and disease prevention,^4^, ^5^, ^6^, ^7^, ^8^, ^9^ TET2 is the only member of the TET family that is frequently mutated in blood stem cells during aging^10^, ^11^, ^12^, ^13^ and whose genetic inactivation drives the onset of myeloid and lymphoid hematological malignancies.^14^, ^15^, ^16^ Previous work has demonstrated that TET2 is recruited to gene regulatory elements, including CpG islands (CGIs) and active enhancers,^17^ and that TET2 loss-of-function mutations result in widespread enhancer DNA hypermethylation in mouse and human hematopoietic cells.^7^^,^^18^, ^19^, ^20^, ^21^ Aberrant enhancer DNA methylation can subsequently alter transcription factor binding specificity, expression of adjacent genes, hematopoietic differentiation trajectories, and may ultimately promote leukemogenesis.^17^^,^^20^^,^^22^, ^23^, ^24^

Recent findings indicate that proline and serine rich 1 (PROSER1) interacts with all 3 TET enzymes and plays a regulatory role in TET-mediated DNA demethylation during development.^25^^,^^26^ PROSER1 was initially identified as an interactor of the ubiquitously transcribed X chromosome tetratricopeptide repeat protein/complex of proteins associated with SET1 complex, specifically interacting with TET2 and O-linked N-acetylglucosamine transferase (OGT) in HEK293 cells.^27^^,^^28^ However, our subsequent studies in mouse embryonic stem cells (mESCs) revealed a broader function for PROSER1 in the assembly of large (∼550 kDa) multiprotein TET complexes. These chromatin-associated complexes, termed TOPD (TET-OGT-PROSER1-DBHS proteins), comprise TET proteins (either TET1, TET2, or TET3), OGT, PROSER1, and dimers of the DBHS protein family (PSPC1, NONO, or SFPQ). Further analyses demonstrated that PROSER1 colocalizes with TET2 at regulatory DNA elements, including CGIs and enhancers, and that depletion of PROSER1 in mESCs disrupts TOPD complexes, impairs TET2 binding at shared loci, and alters DNA methylation patterns.^26^

Considering the pivotal role of TET proteins in the regulation of hematopoiesis and leukemogenesis, it is plausible that PROSER1 and TOPD complexes also modulate DNA methylation patterns in hematopoietic stem and progenitor cells, thereby influencing hematopoiesis. However, the precise role of PROSER1 in hematopoiesis and its relevance to TET-mediated leukemia suppression remains to be elucidated.

## Methods

### Mouse breeding and maintenance

Generation of endonuclease-mediated constitutive PROSER1 knockout (KO) mice is described in a previous study.^26^ To generate constitutive TET2 KO mice, Tet2^fl/fl^^30^ mice were obtained from the Jackson Laboratory (strain number 0175173, B6;129S-TET2^tm1.1laai/J^) and bred to a Cre deleter stain (C57BL/6NTac-*Gt(ROSA)26Sor*^*tm16(cre)Arte*^). CD45.1^+^ mice (B6.SJL- *Ptprc*^*a*^ *Pepc*^*b*^/BoyCrl) were obtained from Charles River (strain code 494). Mouse studies were conducted in accordance with the regulations described in the UK Animals (Scientific Procedures) Act 1986 and were approved by the welfare and ethical use of animals committee of the University of Dundee. Experimental design was in line with the 3Rs principles of replacement, reduction, and refinement.

### Flow cytometry analysis

Freshly harvested bones were crushed using mortar and pestle in 10-mL flow buffer (2% fetal bovine serum [FBS] in phosphate-buffered saline [PBS]; volume-to-volume ratio). Bone marrow (BM) and splenocyte suspensions were passed through 70-μm nylon strainers, spun down, and resuspended in 0.5-mL flow buffer. To analyze peripheral blood, 30 to 40 μL of blood was collected from the tail vein into tubes containing 1 mL of RPMI-1640 with 5% FBS and 5-μM EDTA and mixed immediately. Samples were then erythrolyzed by incubating with 5 mL of ACK buffer (0.15-M NH_4_Cl, 10-mM KHCO_3_, 0.1-mM EDTA, pH 7.2), quenched with 35-mL ice-cold flow buffer, spun, and washed in 5-mL (spleen and BM) or 1-mL (blood) flow buffer. Cultured HOXB8-FL cells were washed and resuspended in flow buffer. The entire blood sample or 1 × 10^6^ to 3 × 10^6^ cells of other cell types were then incubated with 50 μL of Fc block (anti-CD16/32) at 1:50 dilution in flow buffer for 15 minutes, followed by the addition of 50 μL of fluorescently labeled antibody cocktail per million cells at indicated dilutions in the dark for 45 minutes at 4°C. Cells were then washed with flow buffer and resuspended in 300-μL flow buffer containing 0.1 μg/mL 7-aminoactinomycin D (Sigma; A9400). Samples were acquired on NovoCyte NovoSamplerPro flow cytometer (Agilent) and analyzed using FlowJo software (Beckton Dickinson). Sorting of Lin^–^Sca1^+^cKit^+^ (LSK) cells were performed using a MA900 (Sony Biosciences) cell sorter. All centrifugation steps were done in swing-bucket rotors for 5 minutes at 300*g*, 4°C. The details of the antibodies used can be found in supplemental Table 1.

### Adoptive transfer experiments

For primary competitive transplantations, female CD45.1^+^/CD45.1^+^ mice (B6.SJL- *Ptprc*^*a*^ *Pepc*^*b*^/BoyCrl) recipient animals were lethally irradiated (9.5 Gy in 2 equal doses 3 hours apart). Each recipient animal received an IV injection of 8000 sorted LSK cells the following day. These cells were a 1:1 mixture of LSK cells isolated from the following: (1) either wild-type or PROSER1 KO donor mice (CD45.2^+^)^26^; and (2) LSK cells isolated from nonirradiated congenic (CD45.1^+^) donor mice. Chimerism in terminally differentiated hematopoietic lineages was followed by fluorescence-activated cell sorter analysis of peripheral blood samples at 8, 12, 16, and 20 weeks after transplantation. For secondary transplantations, each lethally irradiated recipient animal was IV injected with 6 × 10^6^ unfractionated nucleated BM cells harvested and pooled from primary recipient mice at 20 weeks after transplantation. Hematopoietic tissues were harvested from all recipient animals ∼20 weeks after transplantation and analyzed by flow cytometry. To generate BM chimeric mice, C57BL/6 mice were lethally irradiated (9.5 Gy in 2 equal doses 3 hours apart) and injected IV with 2 × 10^6^ unfractionated nucleated BM cells from wild-type or PROSER1 KO donors the following day. Hematopoietic tissues were collected 11 weeks after transplantation, and LSK cells were sorted for subsequent gene expression analysis.

To assay myeloid leukemogenicity of HOXB8-FL cells lines, 3 × 10^6^ to 4 × 10^6^ freshly AML1-ETO (AE)–transduced HOXB8-FL cells (CD45.2^+^) were mixed with 0.1 × 10^6^ unfractionated nucleated BM support cells (CD45.1^+^) and injected IV into lethally irradiated (9.5 Gy in 2 equal doses 3 hours apart) female CD45.1^+^ mice. Mice were monitored for signs of failed reconstitution and hematological malignancy (anemia, reduced activity, and weight loss) and euthanized at defined time points or earlier if they reached a humane end point. For secondary leukemia cell transplantations, nucleated BM cells isolated as described earlier from the primary diseased animals were cryopreserved on the day of harvest in a (volume-to-volume ratio) solution of 50% FBS, 10% dimethyl sulfoxide, and 40% RPMI at 1 × 10^7^/mL in the vapor phase of liquid nitrogen. After thawing and washing in PBS, 1 × 10^6^ cells in 200-μL PBS were injected into the tail vein of sublethally irradiated (6 Gy in a single dose) female CD45.1^+^ mice. To monitor lymphoid leukemogenicity, 5 × 10^6^ of CRISPR-edited HOXB8-FL cells (PROSER1 KO, TET2 KO, TET3 KO, TET2/TET3 double KO [DKO], or PROSER1/TET2 DKO; CD45.2^+^) were mixed with 0.1 × 10^6^ unfractionated nucleated BM support cells (CD45.1^+^) and injected IV into lethally irradiated (9.5 Gy in 2 equal doses 3 hours apart) female CD45.1^+^ mice. Hematopoietic tissues were harvested from all recipient animals ∼12 weeks after transplantation and analyzed by flow cytometry.

### Generation of mutant HOXB8-FL cell lines

HOXB8-FL cells were derived as previously described and cultured in progenitor outgrowth medium (POM)^29^: RPMI-1640 (Invitrogen; 31870-074) supplemented with 10% mESC-certified FBS (Thermo Fisher Scientific; 16141079), 2-mM l-Glutamine, 0.1-mM β-mercaptoethanol, 100 IU/mL penicillin-streptomycin, 1-μM β-estradiol (E_2_; Sigma; E2758), and 5% filtered cell culture supernatant from a Flt3L-producing B16 melanoma cell line (a kind gift from E. Villablanca). Biallelic disruption of *Proser1* and *Tet3* genes was performed using Alt-R CRISPR-Cas9 system (integrated DNA technologies) according to the manufacturer’s instructions, with ribonucleoprotein complexes containing TrueCut v2 Cas9 protein (Thermo Fisher; A36498), Alt-R CRISPR-Cas9 CRISPR RNA (crRNA) targeting mouse *Proser1* (5′- GTGCTGGATGAAATTCGAA-3′) or *Tet3* (5′-AAGGAGGGGAAGAGTTCTCG-3′) precomplexed with transactivating crRNA (tracrRNA), and carrier single-strand DNA (ssDNA). In brief, these were assembled by annealing equimolar amounts of crRNA and tracrRNA oligos, mixing with equimolar amounts of TrueCut v2 Cas9 protein and ssDNA carrier, and incubating at room temperature for 5 minutes, followed by electroporation into HOXB8-FL cells. Electroporation of HOXB8-FL cells were done using Neon Transfection System 10-μL kit (Invitrogen; MPK1025). Cells were washed in PBS, resuspended in Buffer T, mixed with RNP complexes to the final concentrations of 1 × 10^7^ cells per mL and 1.2-μM RNP complexes, electroporated according to the manufacturer’s instructions using the settings 2200 mV, 10 milliseconds, and 1 pulse, and plated in antibiotic-free POM medium. After a 2-day recovery period in antibiotic-free POM, the cells were resuspended at <500 cells per mL in semisolid E_2_/Flt3L medium (MethoCult M3231 containing 2-mM l-glutamine and 100 IU/mL penicillin-streptomycin, with added 1 μM E_2_ and 5% B16-Flt3L supernatant) and plated onto 35-mm plates at 1 mL per plate. Well-spaced clonal colonies were picked 6 to 7 days after plating under an inverted microscope and cultured in standard POM media for mutation screening.

PROSER1/TET2 DKO lines were generated by biallelic CRISPR-Cas9 disruption of *Proser1* as described earlier in a constitutive TET2 KO line. The TET2/TET3 DKO line was generated by transfecting TET2^fl/fl^/TET3 KO line with StemMACS Cre Recombinase messenger RNA (mRNA; Miltenyi Biotech; 1 × 10^7^ cells per mL and 0.05 μg/μL mRNA), thus inducing excision of exon 3 in the *Tet2* gene locus. The complete deletion of *Tet2* in the transfected population was confirmed by genotyping for *Tet2* alleles^30^ and TET2 western blot at 5 days after transfection. StemMACS mCherry mRNA (130-120-975) was used as a control.

See the supplemental Methods for additional information including immunoprecipitation and mass spectrometry, chromatin immunoprecipitation and RNA sequencing (seq), and DNA methylome analysis.

## Results

### PROSER1 is an integral component of TOPD complexes in hematopoietic cells

**Figure 1.**
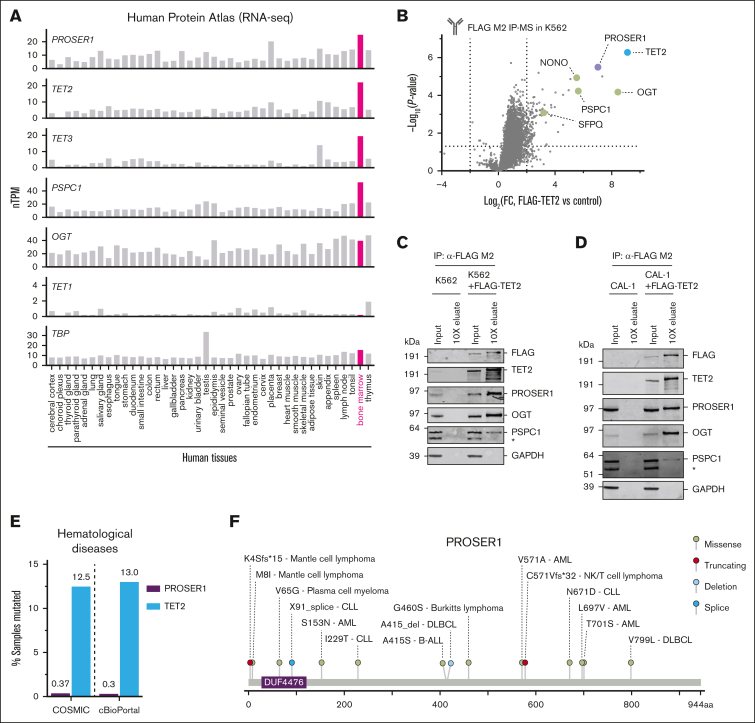
**PROSER1 is an integral component of TOPD complexes in hematopoietic cells.** (A) Bar charts showing Human Protein Atlas normalized mRNA expression (nTPM) across 40 human tissue types for *PROSER1*, *TET2*, *TET3*, *PSPC1*, *OGT*, *TET1*, and *TBP* (reference). All TOPD mRNA transcripts, except *TET1*, are highly expressed in BM. (B) Volcano plot showing protein hits from FLAG M2 immunoprecipitation and mass spectrometry (IP-MS) in K562 cells overexpressing FLAG-tagged full-length human TET2 compared to wild-type control (n *=* 3 biological replicates). Dotted lines indicate fourfold change and *P* value of .05. TOPD complex partners are highlighted and identified. See supplemental Table 2 for full list of enriched proteins in IP-MS experiment. (C) Western blot of input and 10× concentration eluates from FLAG M2 IP on independent lysates from wild-type K562 or K562 cells overexpressing FLAG-tagged full-length human TET2. Asterisk (∗) indicates a nonspecific reactive band. (D) Same as in panel C but for lysates obtained from the human dendritic cell line, CAL-1, overexpressing FLAG-tagged full-length human TET2. (E) Bar chart showing frequency of all PROSER1 and TET2 mutations found in hematological malignancies and listed in COSMIC^33^ and cBioPortal^34^ databases. (F) Lollipop diagram indicating PROSER1 alterations (missense, truncating, deletion, and splice mutations) identified in human hematopoietic malignancies. The change in amino acid sequence as well as disease subtype is shown. Mutational data were curated from COSMIC^33^ and cBioPortal.^34^ AML, acute myeloid leukemia; B-ALL, B-cell acute lymphoblastic leukemia; DLBCL, diffuse large B-cell lymphoma; FC, fold change; NK, natural killer.

To study the role of PROSER1 in hematopoiesis, we initially examined the expression pattern of PROSER1 using a comprehensive mRNA expression atlas of 40 human tissues.^31^^,^^32^ We noted that PROSER1 expression is high in human BM, the main site of hematopoiesis, and mirrors the expression patterns of other TOPD complex members such as TET2, TET3, and PSPC1 (Figure 1A). Furthermore, PROSER1 is constitutively expressed in human hematopoietic stem and progenitor cells as well as myeloid and lymphoid lineages (supplemental Figure 1A). Previous studies have found interactions between PROSER1 and TET proteins in HEK293 cells and mouse mESCs.^26^^,^^28^ To determine whether these interactions are also observed in hematopoietic cells, we expressed FLAG-tagged (DYKDDDDK) full-length TET2 in the human erythroleukemia cell line K562 and determined the TET2 protein-protein interactome. Analysis of triplicate FLAG immunoprecipitation and mass spectrometry experiments revealed significant enrichment of PROSER1 as well as other known TOPD interactors such as OGT and the DBHS proteins PSPC1, NONO, and SFPQ (Figure 1B). These protein-protein interactions could furthermore be validated using immunoprecipitation followed by western blotting (Figure 1C), and similar interactions could be found in the human dendritic cell line CAL-1 (Figure 1D). We also noted that PROSER1 alterations, including missense, splice site, and frameshift mutations, have been identified in various hematological malignancies of both myeloid and lymphoid origin, albeit at much lower frequencies than TET2 mutations (Figure 1E-F). Together, these results suggest that TOPD complex formation is preserved in human hematopoietic cells and that PROSER1 mutations may contribute to disease development.

### The leukemia-suppressive functions of TET proteins are preserved in the absence of PROSER1

Given the critical role of TET proteins in regulating hematopoietic differentiation and prevention of hematological malignancies, we hypothesized that PROSER1 deficiency may mimic the effects of TET2 mutations in hematopoiesis. To test this, we took advantage of a previously described in vitro culture system of primary murine multipotent hematopoietic progenitors based on inducible expression of HOXB8 (HOXB8-FL cells).^29^^,^^35^, ^36^, ^37^ These cells can be maintained indefinitely and, upon withdrawal of E_2_ from the culture media, be instructed to differentiate along myeloid or lymphoid lineages in cell cultures and upon transplantation into mice (supplemental Figure 1B). Using CRISPR-Cas9 gene editing, we isolated independent clonal HOXB8-FL cell lines carrying homozygous frameshift indels in the PROSER1 locus. Furthermore, we isolated HOXB8-FL cell lines with constitutive TET2 KO. Western blotting confirmed complete KO of PROSER1, whereas the expression and stability of TET2 and other TOPD complex partners were unaffected (supplemental Figure 1C-E).

**Figure 2.**
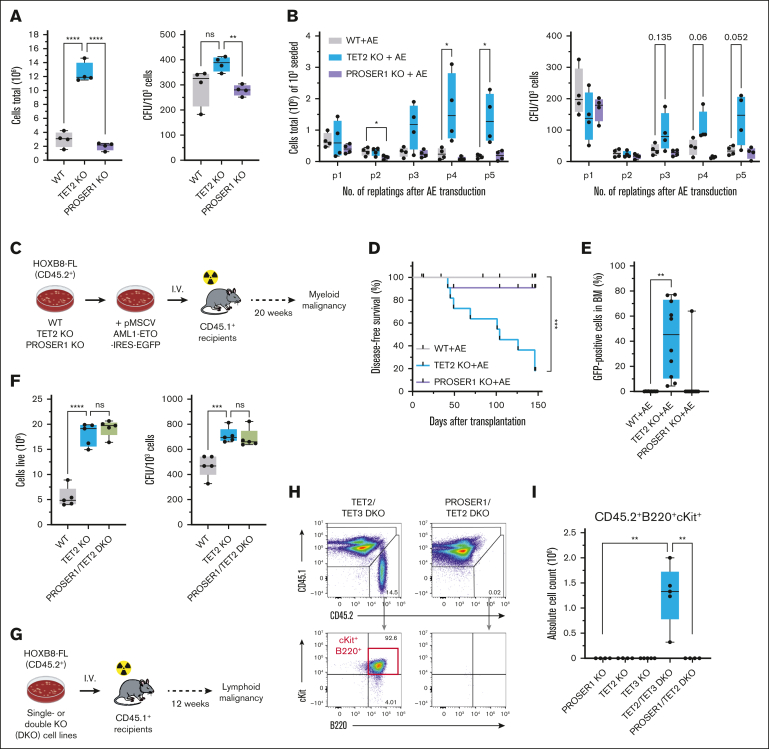
**The leukemia-suppressive functions of TET proteins are preserved in the absence of PROSER1.** (A) Box plots showing cell numbers (left) and colony-forming units (CFUs; right) of wild-type, TET2 KO, and PROSER1 KO HOXB8-FL cells grown in semisolid media (+E_2_ and +Flt3L) for 7 days. Each dot represents an independently derived cell line (n *=* 4). ∗∗*P* < .01; ∗∗∗∗*P* < .0001 (Brown-Forsythe and Welch analysis of variance [ANOVA] test). (B) Same as in panel A but for HOXB8-FL cells expressing AE onco-fusion protein and replated 5 times (7 days each) in semisolid media without E_2_ and with cytokines (+stem cell factor, +interleukin-3 [IL-3], and +IL-6) to support leukemic blast cell growth. ∗*P* < .05 (unpaired 2-tailed *t* test with individual variance for each replating). (C) Schematic illustrating AE in vivo leukemia transplantation assay. (D) Kaplan-Meier plot showing disease-free survival of recipient mice transplanted with AE expressing HOXB8-FL cells of the indicated genotypes. ∗∗∗*P* < .001 (Log-rank [Mantel-Cox] test). (E) Proportion of GFP-positive AE-expressing leukemic blasts in BM of recipient mice at the time of harvest. ∗∗*P* < .01 (unpaired 2-tailed *t* test with Welch correction). (F) Box plots showing cell numbers (left) and CFUs (right) of wild-type, TET2 KO, and PROSER1/TET2 DKO HOXB8-FL cells grown in semisolid media (+E_2_ and +Flt3L) for 7 days. Each dot represents an independently derived cell line (n *=* 4). ∗∗∗*P* < .001; ∗∗∗∗*P* < .0001 (Brown-Forsythe and Welch ANOVA test). (G) Schematic illustrating B-cell malignancy transplantation assay. (H) Representative fluorescence-activated cell sorter (FACS) plots showing expansion of immature B-cell blasts in BM 12 weeks after transplantation of TET2/TET3 DKO HOXB8-FL cells. Immature B-cell blasts are not found in mice receiving HOXB8-FL cells with PROSER1/TET2 DKO as well as PROSER1 KO, TET2 KO, and TET3 KO. (I) Box plot showing absolute counts of CD45.2^+^B220^+^cKit^+^ immature B-cell blasts in BM at 12 weeks after transplantation into lethally irradiated CD45.1 recipient mice. ∗∗*P* < .01 (unpaired 2-tailed *t* test with Welch correction). GFP, green fluorescent protein; ns, nonsignificant; p1, plating no.; WT, wild-type.

We first performed 7-day growth assays in semisolid medium that supports self-renewal activity. Under these conditions, loss of TET2 results in approximately threefold increase in cumulative cell numbers compared to wild-type HOXB8-FL cell lines. In contrast, loss of PROSER1 did not confer enhanced proliferative capacity (Figure 2A). We subsequently expressed the AE oncogene in HOXB8-FL cells and serially replated them in semisolid media under conditions of E_2_ withdrawal (to inhibit HOXB8-driven proliferation) and cytokine supplementation (to promote the growth of leukemic blasts). In accordance with previous findings in lineage-depleted BM cells,^18^^,^^38^ loss of TET2 enhanced the serial replating capacity of AE-transduced HOXB8-FL cells. In contrast, PROSER1 KO cells, such as wild-type cells, steadily lost replating capacity over time (Figure 2B). Finally, we transplanted freshly AE-transduced wild-type, TET2 KO, or PROSER1 KO HOXB8-FL cells into lethally irradiated recipient mice along with CD45.1 support BM cells (Figure 2C). Although most mice that received transplant with TET2 KO cells succumbed to myeloid leukemia, with a median latency of 104 days, only 1 of 12 mice that received PROSER1 KO cells showed signs of distress and was observed to have transduced cells in the BM and spleen at the time of harvest (Figure 2D-E). However, in contrast to cells isolated from a leukemic TET2 recipient mouse, the BM cells from the diseased PROSER1 mouse failed to engraft and cause disease in sublethally irradiated secondary recipient animals, suggesting that these cells were not fully transformed (supplemental Figure 2A-C).

TET3, along with TET2, is highly expressed in BM, and previous reports have established an essential role of these proteins in prevention of lymphoid malignancies.^7^^,^^9^^,^^39^, ^40^, ^41^, ^42^, ^43^, ^44^, ^45^ Therefore, we aimed to test whether loss of PROSER1 alone could drive lymphoid proliferation or play a crucial role when TET activity is low. To investigate this, we took advantage of the robust but transient B-cell differentiation observed when injecting HOXB8-FL cells into recipient mice^29^ and compared cell lines with single-gene KOs or DKOs of PROSER1/TET2 or TET2/TET3. Importantly, deletion of PROSER1 in a TET2 KO background did not reduce the TET2 KO–dependent increase in cell growth and colony-formation capacity (Figure 2F). It is therefore unlikely that PROSER1 KO can suppress the effects of TET2 loss of function in hematopoietic progenitors. We then transplanted these cells into lethally irradiated recipient mice alongside CD45.1^+^ support BM cells and harvested hematopoietic tissues 12 weeks after transplant (Figure 2G). Notably, we found that concurrent deletion of TET2 and TET3 in HOXB8-FL cells results in an early blockade of B-cell differentiation, characterized by an expansion of immature CD45.2^+^B220^+^cKit^+^ blast cells within the BM of recipient mice. Conversely, mice receiving HOXB8-FL cells with individual deletions of PROSER1, TET2, or TET3 exhibit minimal or no presence of CD45.2^+^ cells, indicative of exhaustion of HOXB8-FL repopulation potential at this late time point. Finally, the combined deletion of TET2 and PROSER1 failed to induce a B-cell differentiation block, suggesting that, despite the loss of both TET2 and TOPD complexes, the residual TET3 catalytic activity is sufficient to suppress the expansion of aberrant B-cell progenitors (Figure 2H-I; supplemental Figure 2D-F). Overall, these results demonstrate that loss of PROSER1 is insufficient to induce aberrant proliferation and promote myeloid or lymphoid transformation via dysregulation of TET-mediated epigenetic regulation.

### PROSER1 loss leads to aberrant DNA methylation in hematopoietic progenitors

**Figure 3.**
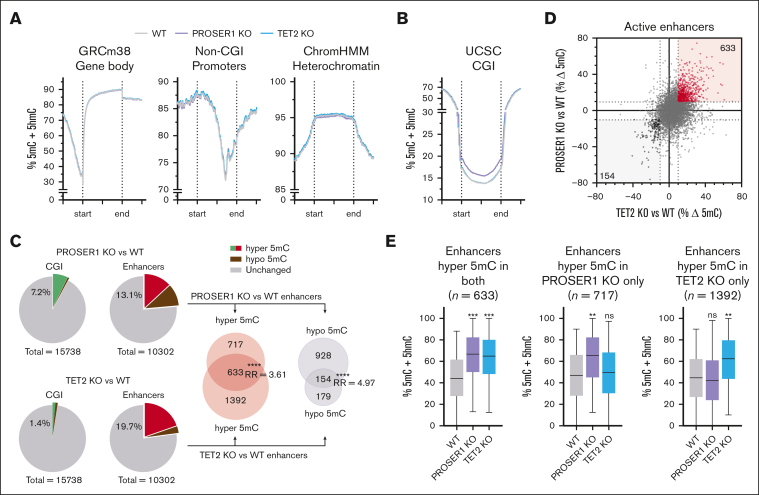
**PROSER1 loss leads to aberrant DNA methylation in hematopoietic progenitors.** (A) Quantitation trend plots of DNA methylation quantified by enzymatic methyl sequencing (EM-seq) in gene bodies, non-CGI promoters, and heterochromatin for CpG sites covered by minimum 10 EM-seq reads in all samples in WT, PROSER1 KO, and TET2 KO HOXB8-FL cells. (B) Same as in panel A but showing DNA hypermethylation in CGIs in PROSER1-deficient HOXB8-FL cells. (C) Pie charts showing fraction of hypermethylated (>10% average increase) or hypomethylated (>10% average decrease) CGIs and active enhancers as well as the overlap of active enhancers with altered DNA methylation in PROSER1 KO and TET2 KO HOXB8-FL cells. ∗∗∗∗*P* < .0001 (2-tailed Fisher exact test). (D) XY scatterplot showing correlation between DNA methylation changes at active enhancers in TET2 (horizontal x-axis) and PROSER1 (vertical y-axis) KO HOXB8-FL cells. Each dot represents average methylation difference of all CpG sites (>10 reads) within a specific enhancer. (E) Box plots showing average enhancer DNA methylation in wild-type, PROSER1 KO, and TET2 KO HOXB8-FL cells at enhancers with >10% hypermethylation in both genotypes (left), PROSER1 KO only (middle), or TET2 KO only (right) cells. Error bars represent the ±min/max, and the line is the median. The effect sizes of DNA methylation change compared to wild-type was measured with Cohen *d*. ∗*d* > 0.3 (small effect); ∗∗*d* > 0.6 (medium effect); ∗∗∗*d* > 0.9 (large effect). max, maximum; min, minimum; ns, nonsignificant; RR, relative risk; WT, wild-type; UCSC, University of California Santa Cruz.

Although PROSER1 deficiency does not appear to contribute to the development of TET-related hematological malignancies, it is likely to modulate DNA demethylation and potentially have additional functions in regulation of hematopoiesis. To gain more insight into this, we generated base-resolution DNA methylation profiles using enzymatic methyl sequencing from independently derived wild-type, PROSER1 KO, and TET2 KO HOXB8-FL cell lines, as well as genome-wide maps of histone modifications H3K4me1, H3K27ac, and H3K27me3 to define the activation status of promoters and enhancers (supplemental Figure 3A-B). In contrast to the global DNA hypomethylation phenotype previously reported in PROSER1-deficient mESCs,^26^ the overall genomic DNA methylation landscape, including gene bodies, non-CGI promoters, and heterochromatic domains, remained largely unchanged in PROSER1-deficient hematopoietic progenitor cells (Figure 3A). Conversely, a marked increase in DNA methylation was observed at CGIs, with 1127 CGIs exhibiting a >10% average increase in DNA methylation (Figure 3B-C). Enrichment analysis of gene ontology terms revealed that most hypermethylated CGIs were associated with genes encoding developmental regulators (supplemental Figure 3C), aligning with the proposed role of PROSER1 in early developmental processes.^26^ These genes were furthermore found to be enriched for H3K27me3 and typically silenced in both wild-type and PROSER1-deficient HOXB8-FL cells (supplemental Figure 3D-E). Together, this suggests that the observed CGI hypermethylation may only have a minor impact on hematopoietic gene expression programs.

Analysis of DNA methylation changes at active enhancer regions also uncovered substantial alterations. Using a threshold of >10% average DNA methylation gain or loss, we identified 1350 hypermethylated and 1082 hypomethylated enhancers in PROSER1 KO cells (Figure 2C). Gene ontology analysis indicated that enhancers exhibiting both increased and decreased DNA methylation were associated with genes important for hematopoietic differentiation (supplemental Figure 3F-G). In contrast, loss of TET2, as previously reported in both murine and human hematopoietic cells,^7^^,^^17^, ^18^, ^19^, ^20^, ^21^ resulted predominantly in increased enhancer DNA methylation affecting ∼20% of all active enhancers (Figure 3C). Interestingly, comparative analysis of enhancer methylation patterns in PROSER1- and TET2-deficient cells identified a significant subset of enhancers (*P* < .0001; Fisher exact test) that were hypermethylated upon loss of either protein (Figure 3C-D). This shared subset of 633 hypermethylated enhancers exhibited similar increases in DNA methylation and accounted for approximately one-third of the total enhancer hypermethylation observed in TET2-deficient cells (Figure 3E). In addition, we observed enhancer hypermethylation of a unique set of 717 enhancers in PROSER1-deficient cells, potentially due to the concurrent impairment of TET3 function (Figure 3E). Collectively, these data indicate a potential cooperative function of PROSER1 and TET2 and possibly other TOPD complex members, including TET3, in maintaining low DNA methylation at CGIs and enhancers, which may affect normal hematopoiesis.

### PROSER1 deficiency impairs regulation of genes involved in cell cycle and HSC homeostasis and leads to exhaustion of HSC activity upon serial transplantations

**Figure 4.**
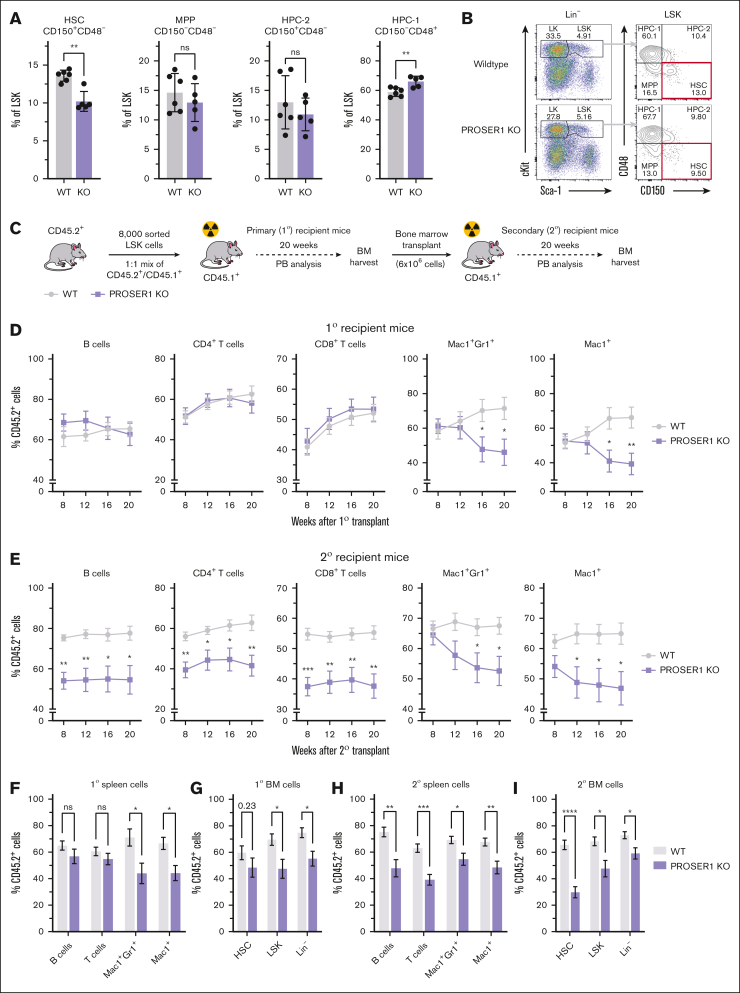
**Loss of PROSER1 leads to exhaustion of HSC activity upon serial transplantations.** (A) Bar charts showing fraction of CD150^+^CD48^–^ long-term HSCs, CD150^–^CD48^–^ MPP, CD150^+^CD48^+^ HPC-2, and CD150^–^CD48^+^ HPC-1 cells within the LSK population in 5- to 6-month-old wild-type and PROSER1-deficient animals. Data represent mean ± standard deviation (n = 5-6). ∗∗*P* < .01 (unpaired 2-tailed *t* test with Welch correction). (B) Representative FACS plots showing gating strategy^46^ as well as CD150/CD48 surface marker expression in LSK cells harvested from wild-type and PROSER1-deficient animals. (C) Schematic showing serial transplantation assay. Sorted LSK cells from CD45.2^+^ wild-type or PROSER1 KO mice were mixed with equal numbers of sorted CD45.1^+^ LSK competitor cells and transplanted into lethally irradiated primary (1°) CD45.1^+^ recipients. After 20 weeks, 6 × 10^6^ unfractionated nucleated BM cells pooled from primary mice were retransplanted into lethally irradiated secondary (2°) recipient mice. (D) Percentages of CD45.2^+^ cells in periodic peripheral blood samples from 1° recipient mice in B220^+^ B-cell, CD4^+^ T-cell, CD8^+^ T-cell, Mac1^+^Gr1^+^ neutrophilic granulocyte, and Mac1^+^ monocyte/granulocyte cell populations over 20 weeks. Data represent mean ± standard error of the mean (SEM) (n = 8). ∗*P* < .05; ∗∗*P* < .01 (unpaired 2-tailed *t* test with Welch correction). (E) Same as in panel D but for 2° recipient animals. (F,H) Bar charts showing percentages of CD45.2^+^ cells in the indicated terminal differentiated lineages in spleens harvested from 1° (F) or 2° recipient animals (H) after 20 weeks. (G,I) Same as for panels F,H but for hematopoietic stem and progenitor cells in BM harvested from 1° (G) or 2° recipient animals (I) after 20 weeks. Data represent mean ± SEM (n = 8). ∗*P* < .05; ∗∗*P* < .01; ∗∗∗*P* < .001; ∗∗∗∗*P* < .0001 (unpaired 2-tailed *t* test with Welch correction). HPC, hematopoietic progenitor cell; MPP, multipotent progenitor; ns, not significant; PB, peripheral blood; WT, wild-type.

Encouraged by these findings, we sought to investigate the impact of PROSER1 deletion on hematopoietic stem cell (HSC) function and lineage differentiation using a previously described constitutive PROSER1 KO mouse model.^26^ Analysis of terminally differentiated lineages and progenitor cells in adult PROSER1-deficient mice (aged ∼5-6 months) revealed that the hematopoietic system was largely unaffected under steady-state conditions (supplemental Figure 4A-E). However, detailed analyses of the stem and progenitor compartment showed a decrease in the proportion and absolute number of long-term HSCs (CD150^+^CD48^–^) within the LSK population (Figure 4A-B; supplemental Figure 4F). To investigate this further, we performed serial competitive transplantation assays using CD45.1/CD45.2 congenic markers to test the cell-autonomous effect of PROSER1 deficiency on long-term reconstitution and multilineage differentiation capabilities (Figure 4C). Peripheral blood analysis of primary recipient mice demonstrated that LSK cells from wild-type littermates showed robust long-term reconstitution of both myeloid and lymphoid lineages. In contrast, LSK cells from PROSER1 KO mice, although initially exhibiting strong repopulation, showed a significant decline in myeloid lineage output at 16 weeks after transplantation (Figure 4D). In line with these findings, PROSER1 KO cells had reduced contribution to myeloid lineages within the spleens of recipient animals at the 20-week end point (Figure 4F). Additionally, we observed a modest decrease in PROSER1 KO HSCs and downstream progenitors within the BM (Figure 4G), suggesting an emerging deficiency in the stem cell compartment that is initially manifested in myeloid cell types due to their relative rapid turnover. To investigate this further, we transplanted unfractionated pooled BM from primary recipients into lethally irradiated secondary recipient animals (Figure 4C). Analysis of periodic peripheral blood samples revealed a decrease in the ability of PROSER1 KO cells to reconstitute multilineage hematopoiesis, affecting both myeloid and lymphoid lineages (Figure 4E). This was furthermore accompanied by a reduction in contribution of PROSER1 KO cells to all major terminally differentiated blood lineages within the spleen of secondary recipient animals, as well as a sharp decrease of PROSER1 KO long-term HSCs in the BM at the 20-week end point (Figure 4H-I).

**Figure 5.**
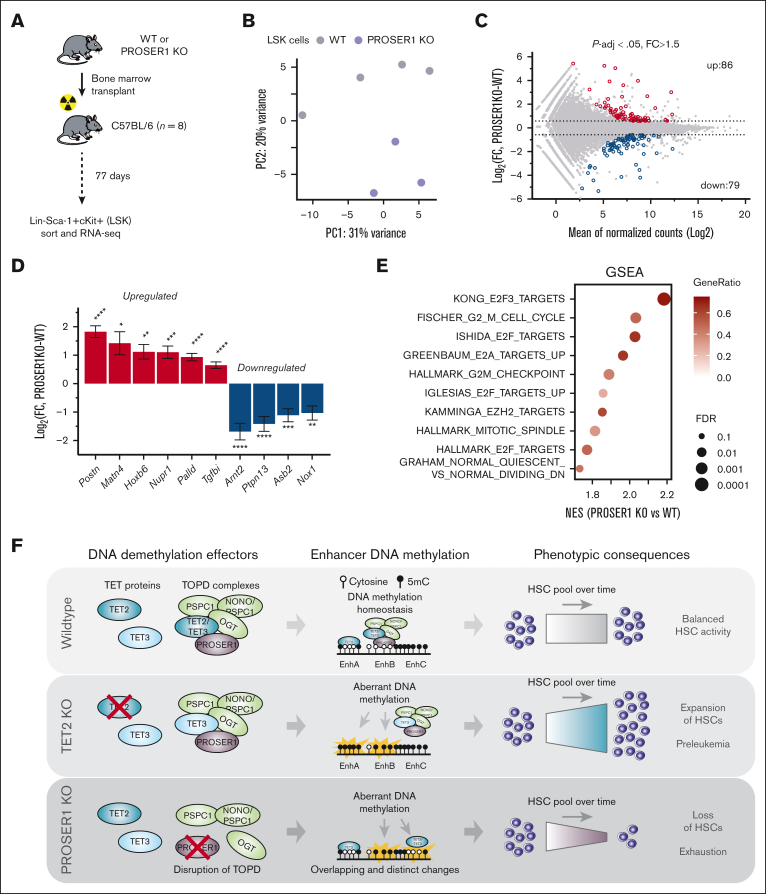
**Deregulation of genes involved in cell cycle and HSC homeostasis in PROSER1-deficient LSK cells.** (A) Schematic showing experimental setup to isolate wild-type and PROSER1-deficient LSK cells from BM chimeric animals. C57BL/6 recipients were lethally irradiated. (B) Principal component analysis plot of RNA seq data from biological replicate samples of wild-type (n = 4) and PROSER1 KO (n = 3) LSK cells. (C) MA plot showing differential expression analysis of wild-type and PROSER1 KO LSK cells. Red and blue symbols indicate significantly differentially upregulated and downregulated genes, respectively (threshold, *P*-adj < .05; FC > 1.5). The total number of differentially regulated genes in each category are indicated. See supplemental Table 3 for full list of differentially expressed genes. (D) Differential expression of selected genes with known roles in HSC homeostasis. Bars represent log_2_ fold change of expression in PROSER1 KO relative to wild-type LSK. Error bars represent the estimated standard error from DESeq2 output. Statistical significance was measured by Benjamini-Hochberg adjusted *P* value. ∗*P*-adj < .05; ∗∗*P*-adj < .01; ∗∗∗*P*-adj < .001; ∗∗∗∗*P*-adj < .001. (E) Enrichment scores (GSEA) of PROSER1 KO vs wild-type LSK cells in gene sets associated with cell cycle regulation and HSC quiescence. NES, gene ratios, and –log_10_ FDR values are displayed for each gene signature. (F) Illustration of the roles of PROSER1 and TET2 in regulation of DNA methylation and hematopoiesis. PROSER1 is a pan-TET interactor important for maintenance of DNA methylation homeostasis in hematopoietic cells. Loss of PROSER1 results in disruption of TOPD complexes and overlapping and distinct enhancer DNA methylation changes compared to changes observed in TET2 KO cells. These DNA methylation changes and the resulting deregulation of genes governing cell cycle and HSCs likely drive the observed exhaustion of HSC activity over time. GSEA, gene set enrichment analysis; FDR, false discovery rate; FC, fold change; NES, normalized enrichment scores; WT, wild-type.

To further investigate the primary and cell-autonomous molecular consequences of loss of PROSER1 in hematopoietic stem and progenitor cells, we transplanted wild-type or PROSER1 KO BM into lethally irradiated recipients and performed RNA seq in LSK cells harvested at day 77 after transplant, a time point before the onset of the PROSER1 phenotype (Figure 5A). Computational analysis revealed distinct clustering of the wild-type and KO samples (Figure 5B), as well as 165 differentially expressed genes (*P*-adj < .05; fold change > 1.5; Figure 5C). Among these, we observed upregulation of Periostin (*Postn*),^47^ Matrilin-4 (*Matn4*),^48^ Nuclear Protein 1, Transcriptional Regulator (*Nupr1*),^49^ and Cyclin-Dependent Kinase Inhibitor 2C (*Cdkn2c*),^50^ as well as downregulation of Aryl Hydrocarbon Receptor Nuclear Translocator 2 (*Arnt2*)^51^ and Protein Tyrosine Phosphatase Non-Receptor Type 13 (*Ptpn13*),^52^ all of which are implicated in the regulation and function of HSCs (Figure 5D). Analysis of enriched gene signatures furthermore revealed that PROSER1 deficiency results in increased expression of cell cycle–related genes as well as high expression of genes that are normally downregulated in quiescent CD34^+^ HSCs compared to activated CD34^+^ HSCs (Figure 5E). Taken together, these results suggest that PROSER1 deficiency disrupts the balance between HSC activation and quiescence, potentially caused by aberrant expression of specific HSC regulatory genes. This imbalance leads to increased cell cycle entry and a subsequent exhaustion of HSC activity, initially evident as reduced myeloid lineage differentiation and ultimately resulting in a compromised capacity for multilineage hematopoietic reconstitution (Figure 5F).

## Discussion

The prevalent occurrence of mutations in DNA methylation effectors, such as TET2 and DNMT3A, in individuals with clonal hematopoiesis^10^, ^11^, ^12^, ^13^ suggests that HSCs are particularly sensitive to disruption of DNA methylation homeostasis. It is therefore conceivable that mutations not only in the DNA methylation regulators themselves but also in accessory proteins that modulate their function, such as PROSER1, can lead to dysregulated hematopoiesis. Here, we show that loss of PROSER1, in comparison to TET2 loss, causes overlapping and distinct changes to the DNA methylome of hematopoietic progenitor cells. Specifically, we identified a subset of enhancers that exhibit hypermethylation upon loss of either TET2 or PROSER1, suggesting a cooperative relationship between these proteins at these specific genomic loci. Thus, although TET2 is likely to directly interact with many active enhancers through other mechanisms, our data suggest that TET2 is recruited to, as well as prevents aberrant DNA methylation at, a subset of enhancers via formation of PROSER1-dependent TOPD complexes. Of note, loss of PROSER1 also leads to reduced DNA methylation at hematopoiesis-associated enhancers that are not affected by TET2 KO (Figure 3C; supplemental Figure 3G). Although this could be attributed to indirect effects, potentially arising from altered gene expression after PROSER1 KO, it may also result from the release of TET2 and/or TET3 from TOPD complexes upon PROSER1 loss. This mechanism, similar to that observed in mESCs,^26^ could lead to excessive DNA demethylation at enhancers where TET chromatin recruitment is not directly dependent on PROSER1 and TOPD complexes. Due to technical difficulties in solubilizing TET2-DNA complexes in HOXB8-FL chromatin, TET2 chromatin immunoprecipitation-seq analysis was unsuccessful. Consequently, we were unable to generate a reliable TET2 genome occupancy data set in wild-type and PROSER1-deficient HOXB8-FL cells to directly test this hypothesis.

Despite exhibiting a partially overlapping DNA methylation phenotype, our findings show that PROSER1 does not phenocopy loss of TET2 in the development of myeloid or lymphoid hematological malignancies. In contrast, we observed that PROSER1 deficiency leads to deregulation of genes involved in cell cycle and HSC homeostasis, exhaustion of HSC activity, and impaired multilineage hematopoietic reconstitution. This phenotype is opposite to the observed increase in stem cell renewal and age-related myeloid bias reported in investigations of TET2-deficient hematopoiesis.^30^^,^^42^^,^^53^, ^54^, ^55^ Interestingly, a recent study found that *Tie2*-Cre–mediated TET3 deletion impairs HSC homeostasis and results in HSC exhaustion in adult mice.^56^ This phenotypic similarity suggests that the PROSER1 KO phenotype may, at least partly, result from loss of TET3 function in HSCs. However, the specific roles of the TET family members in the observed PROSER1- and TOPD-dependent impairment of HSC function require further investigation. Given that other TOPD-interacting factors, such as OGT and DBHS proteins, likely have functions beyond their involvement in the TOPD complexes, their specific TOPD-dependent role in maintaining HSC function remains unclear.

In summary, our findings indicate that TET-mediated DNA demethylation can both restrict HSC expansion (through TET2-mediated catalytic activity and suppression of stem cell activity) but is also important to sustain normal hematopoiesis (via TET protein integration in PROSER1-dependent TOPD complexes). Although PROSER1 loss results in overlapping enhancer DNA hypermethylation, affecting approximately one-third of the enhancers affected by TET2 loss, the lack of stem cell expansion suggests that the critical enhancers driving this phenotype are likely among the remaining two-thirds of enhancers hypermethylated due to TET2 deficiency. Conversely, PROSER1-dependent DNA methylation changes in either hypermethylated or hypomethylated enhancers or both may contribute to the loss of HSC activity. However, the specific roles of epigenetic dysregulation of these enhancer subsets in the observed phenotypes require further investigation.

In closing, we propose PROSER1 as a cofactor of TET enzymes that is important for the maintenance of HSC function. Our work sets the stage for further investigations focusing on how PROSER1 deficiency and the subsequent dysregulation of DNA methylation may affect the function of terminally differentiated blood cells, as well as its contribution and potential synergy with other oncogenic hits toward the development of hematological diseases.

Conflict-of-interest disclosure: The authors declare no competing financial interests.

The current affiliation for A.F. is Department of Medical and Molecular Genetics, Faculty of Life Sciences and Medicine, King’s College London, London, United Kingdom.

## Supplementary Material

Supplemental Figures, Methods, and References
