# Supplementary material for: The TET protein family interactor PROSER1 sustains hematopoietic stem cell function
Source: Blood Adv. 2025 Jun 26;9(17):4378–90. doi: 10.1182/bloodadvances.2024015683 (PMC12447449; doi:10.1182/bloodadvances.2024015683)
Supplement: Supplemental Figures, Methods, and References [file BLOODA_ADV-2024-015683-mmc1.pdf]

The TET protein family interactor PROSER1 sustains hematopoietic stem cell function

Elena V. Knatko[1], Anna Fleming[1,2], Xiang Li[1], Phoebe Crawley[1], Ieva Budriunaite[1], Kasper D. Rasmussen[1]

[1]Division of Molecular, Cellular, and Developmental Biology, University of Dundee, Dundee DD1 5EH, UK.
[2]present address: Department of Medical and Molecular Genetics, Faculty of Life Sciences and Medicine, King's College London, London SE1 9RT, UK.

Corresponding author: k.d.rasmussen@dundee.ac.uk

CONTENT

Supplemental Figure 1
Supplemental Figure 2
Supplemental Figure 3
Supplemental Figure 4
SUPPLEMENTARY METHODS
SUPPLEMENTARY REFERENCES

Supplemental Fig. S1

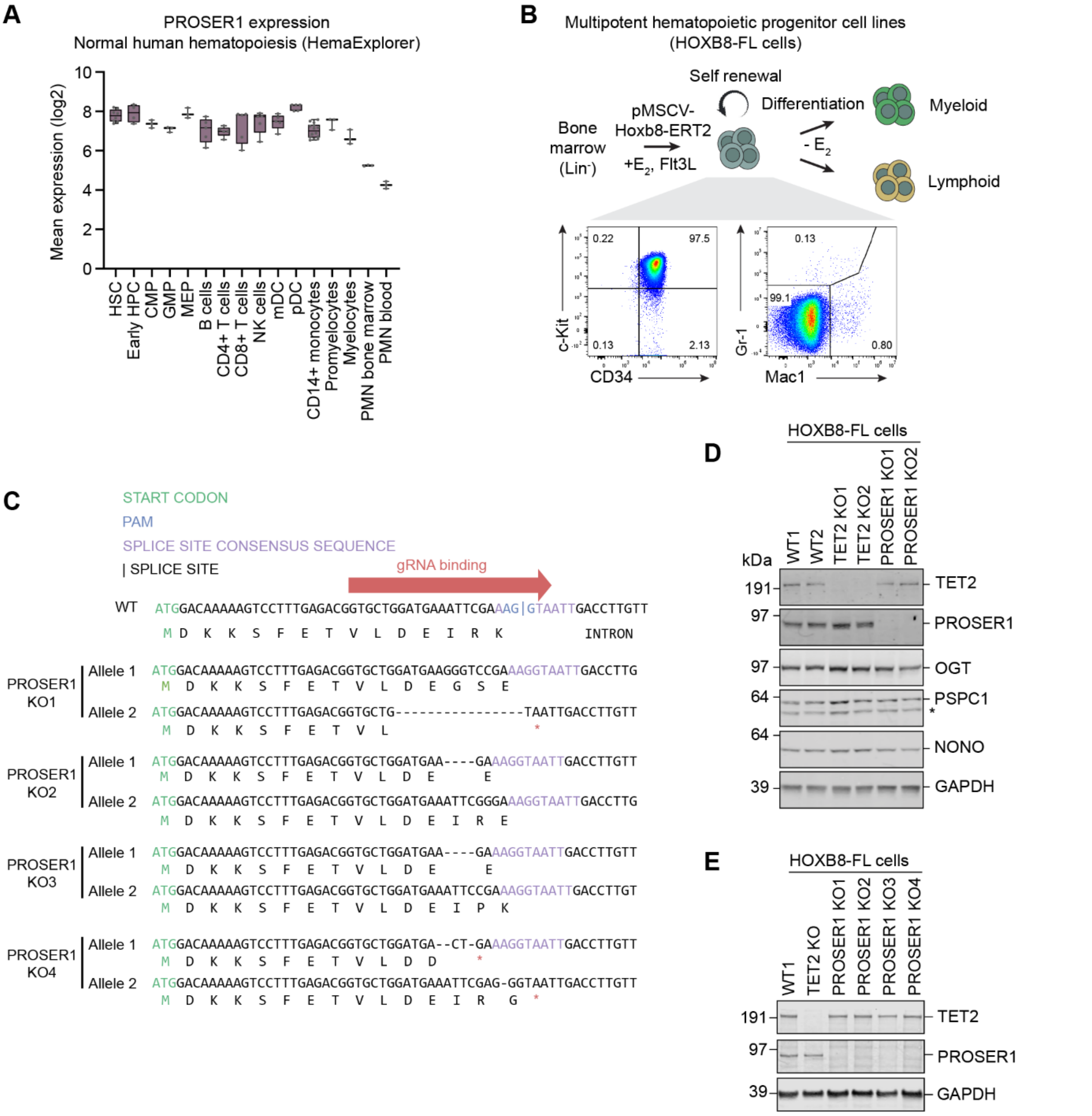

Supplemental Fig. S1. A. Expression of PROSER1 in human hematopoietic cell types. Expression data was downloaded from BloodSpot 3.0 [1] and plotted to show the normalised intensity of the 225887_at probeset in the HemaExplorer dataset. **B.** Illustration of *in vivo* and *in vitro* differentiation potential of multipotent HOXB8-FL progenitors [2] and their surface marker expression during self-renewal conditions (β-Estradiol (E_2) and Flt3 ligand (Flt3L)). **C.** Insertions and deletions in the N-terminus of *Proser1* gene in the four PROSER1 knockout HOXB8-FL lines used in this study. For each clone both alleles are represented, along with the peptide sequences. **D.** and **E.** Western blot validation of TET2 knockout and PROSER1 knockout HOXB8-FL cell lines. * indicates a non-specific reactive band.

Supplemental Fig. S2

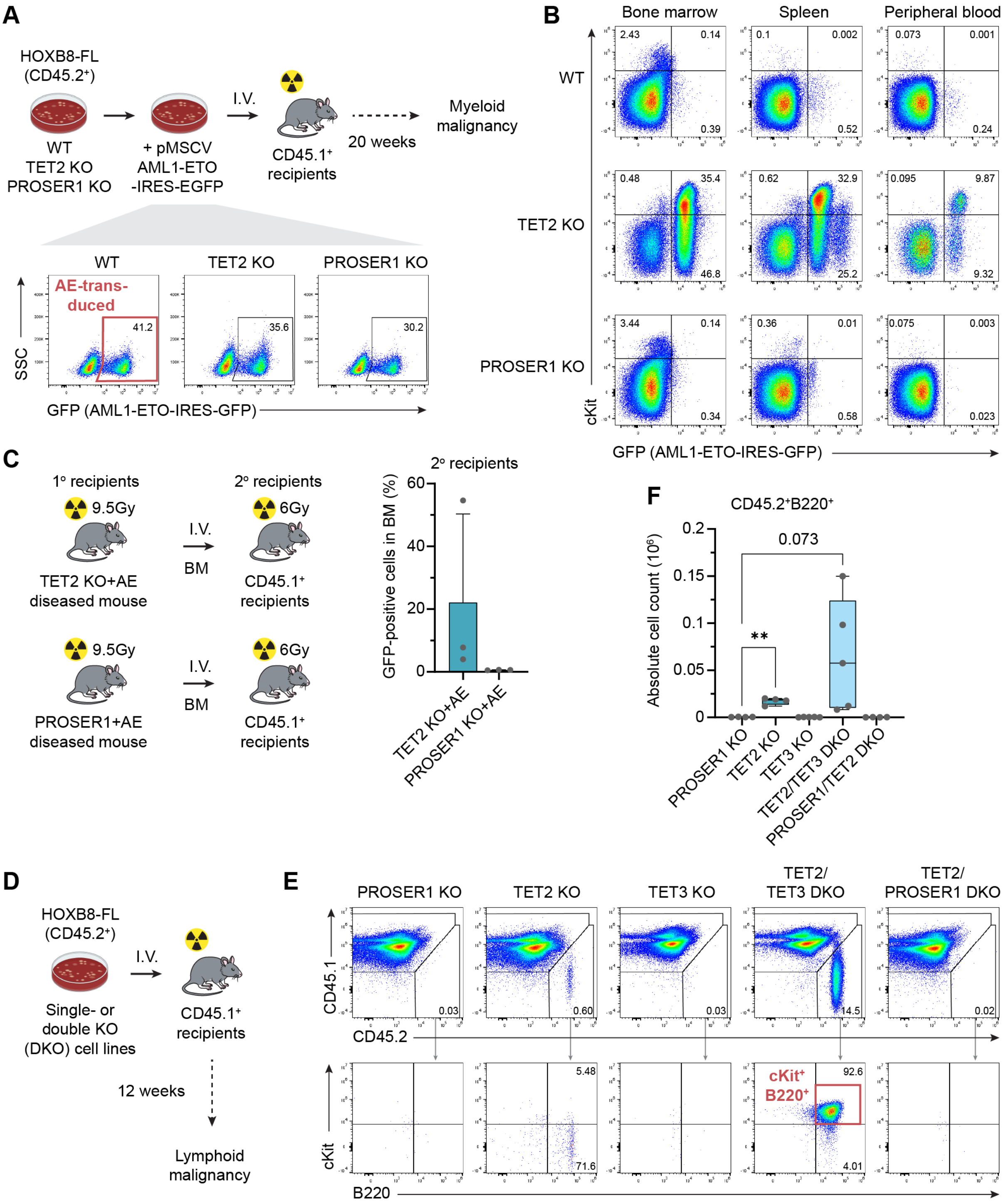

Supplemental Fig. S2. A. Schematic illustrating AML1-ETO in vivo leukemia transplantation assay. Lethally irradiated mice were injected with 4-5x10^6 AE-transduced HOXB8-FL cells mixed with 1x10^5 CD45.1$^+$ nucleated bone marrow support cells. Representative FACS plots showing AML1-ETO-IRES-GFP expression at the time of transplantation is presented **B.** Representative FACS plots showing surface marker expression of AML blast cells isolated from bone marrow, spleen and peripheral blood

of a recipient mice transplanted with TET2 knockout + AML1-ETO HOXB8-FL cells. Blast cells express EGFP and cKit. **C.** Schematic (left) and bar chart showing proportion of GFP-positive AE-expressing leukemic blasts (right) in bone marrow of sublethally (6 Gy) irradiated secondary (2°) recipient mice (*n* = 3) at the time of harvest. **D.** Schematic illustrating B cell malignancy transplantation assay. Lethally irradiated mice were injected with 5×10^6 HOXB8-FL cells mixed with 1×10^5 CD45.1 nucleated bone marrow helper cells. **E.** Representative FACS plots showing surface marker expression of immature B cell blasts ($CD45.2^+B220^+cKit^+$) in the bone marrow of recipient mice transplanted with TET2/TET3 DKO HOXB8-FL cells. Mice transplanted with TET2 knockout cells show a modest expansion of $CD45.2^+B220^+$ cells whereas all other genotypes fail to contribute to the B cell lineage likely due to exhaustion of repopulating capacity. **F.** Box plot showing absolute counts of CD45.2+B220+ B cells in bone marrow at 12 weeks after transplantation into lethally irradiated CD45.1 recipient mice. ** p<0.01, unpaired two-tailed *t*-test with Welch's correction.

Supplemental Fig. S3

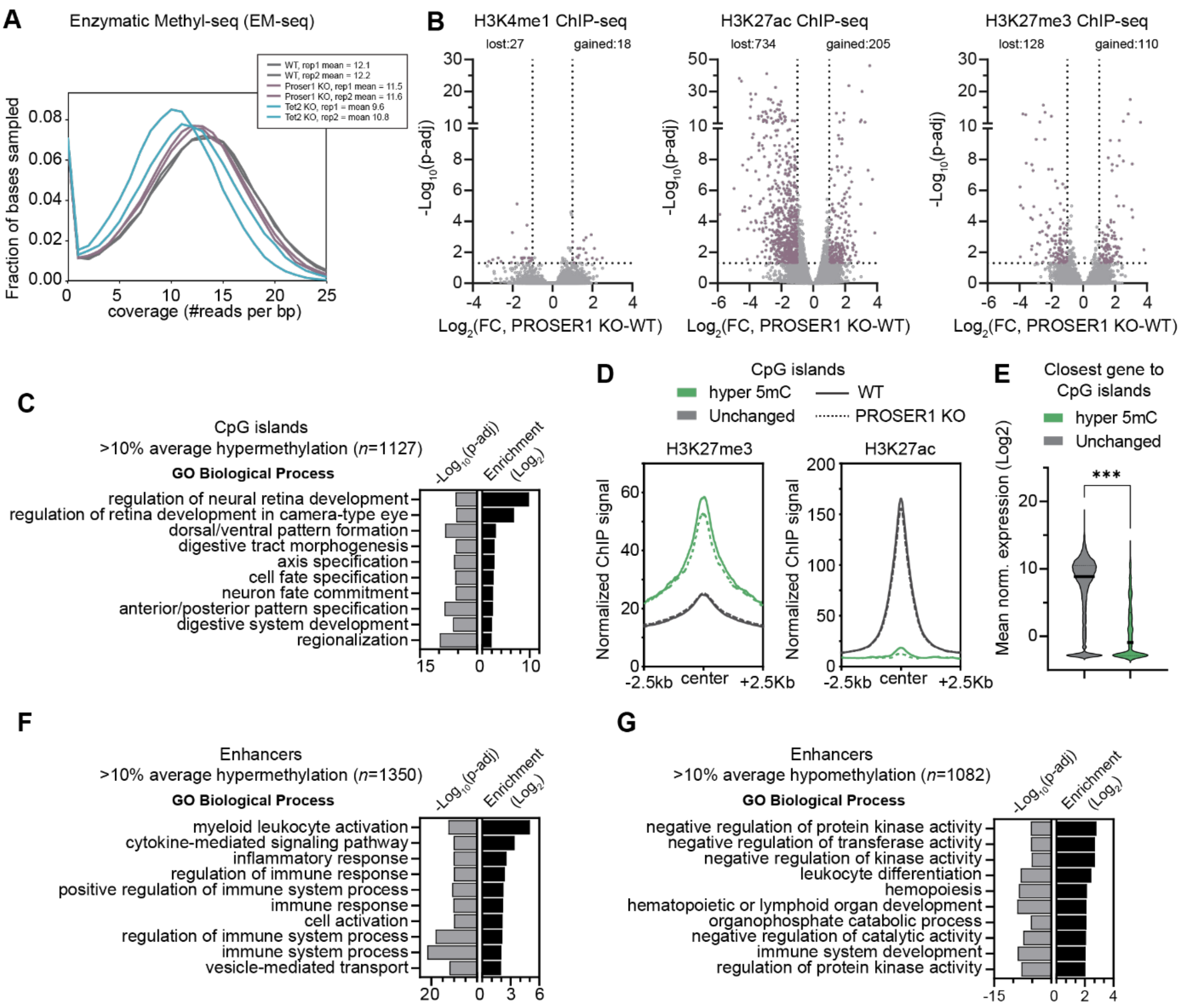

Supplemental Fig. S3. A. Average EM-seq read coverages in independent wildtype, TET2 knockout and PROSER1 knockout HOXB8-FL cell lines. The replicates within each genotype were pooled for subsequent analyses shown in main figure requiring high read coverage at individual sites. **B.** Volcano plot showing changes in H3K4me1 (left) (*n*=3 biological replicates), H3K27ac (center) (*n=2* biological replicates) or H3K27me3 (right) (*n=2* biological replicates) enrichment on chromatin identified by ChIP-seq in WT and PROSER1 knockout HOXB8-FL cells. Dotted lines indicate 2-fold change and p-adj 0.05. Sites where p-adj<0.05 and Abs(fold change≥2) are highlighted and their number in each quadrant is given. **C.** Bar charts showing enriched gene ontology (GO) terms identified by GREAT [3] for CGIs with greater than 10% average hypermethylation (minimum 10 EM-seq reads per CpG in all samples) in PROSER1 knockout HOXB8-FL cells compared to wildtype. **D.** Line plot showing mean H3K27me3 (left) and H3K27ac (right) ChIP signal at hypermethylated (*n* = 1,127) and unchanged CGIs (*n* = 14,882) in wildtype (solid line) and PROSER1 knockout (dashed line) HOXB8-FL cells. **E.** Violin plot showing average mRNA expression of genes closest to unchanged CGIs, or genes closest to hypermethylated CGIs. Genes associated with both hypermethylated and unchanged CGIs were excluded to remove mixed effects on gene expression. The effect sizes of DNA methylation change compared to wildtype was measured with Cohen's d. * d>0.3 (small effect), ** d>0.6 (medium effect), *** d>0.9 (large effect). **F.** Bar charts showing enriched gene ontology (GO) terms identified by GREAT [3] ('Basal plus extension') for genes associated with active enhancers with greater than 10% average hypermethylation (minimum 10 EM-seq reads per CpG in all samples) in PROSER1 knockout HOXB8-FL cells compared to wildtype. **G.** same as **F.**, but for genes associated with enhancers with greater than 10% average hypomethylation in PROSER1 knockout HOXB8-FL cells compared to wildtype.

Supplemental Fig. S4

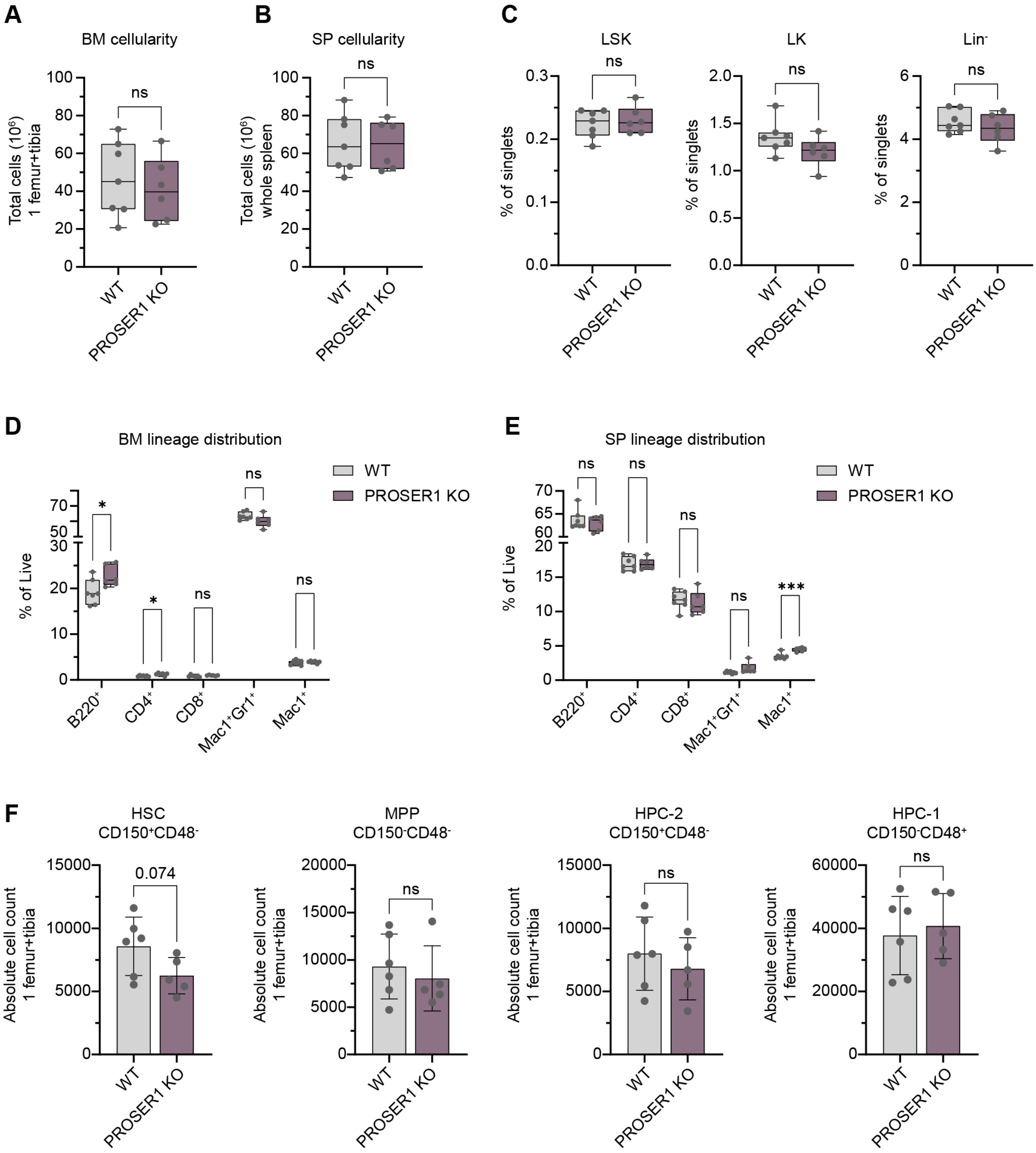

Supplemental Fig. S4. A. Box plots showing total number of cells in bone marrow (one femur+tibia) harvested from 20-24 weeks old wildtype or PROSER1 knockout animals (*n* = 6-7). Error bars represent the ±min/max and line is the median. Statistical significance was measured using unpaired two-tailed *t*-test with Welch's correction. ns, non-significant. **B.** same as **A.**, but for cells harvested from whole spleen. **C.** Percentages of hematopoietic progenitors in bone marrow harvested from 20-24 weeks old wildtype and PROSER1 knockout animals (*n* = 6-7). Individual box plots show $Lin^-Sca1^+cKit^+$ (LSK) (left), Lin^-cKit^+ (LK) (middle), or Lin^- (right) populations. **D.** Same as for **C.**, but for terminally differentiated $B220^+$ B cells, $CD4^+$ T-cells, $CD8^+$ T-cells, $Mac1^+Gr1^+$ neutrophilic granulocytes and $Mac1^+$

monocytes/granulocytes cells in bone marrow. **E.** Same as for **D.**, but for terminally differentiated cells in the spleen. * $p<0.05$ unpaired two-tailed *t*-test with Welch's correction. ns, non-significant. **F.** Bar charts showing absolute numbers of $CD150^+CD48^-$ long-term HSCs, $CD150^-CD48^-$ MPP, $CD150^+CD48^+$ HPC-2, and $CD150^-CD48^+$ HPC-1 cells within the $Lin^-Sca1^+cKit^+$ (LSK) population in 5-6 months old wildtype and PROSER1-deficient animals from 1 femur and tibia (related to Fig. 4A). Data represent mean ± SD (*n*=5-6). Statistical significance was measured using unpaired two-tailed *t*-test with Welch's correction. ns, not significant.

SUPPLEMENTARY METHODS

Derivation and growth of HOXB8-FL cells

HOXB8-FL cells were derived as previously described [2]. Phoenix-Eco cells were transfected with the MSCV-Neo-ERHBD-HOXB8 vector, a retroviral expression vector containing mouse HOXB8 protein fused to the human estrogen receptor (A kind gift from H. Haecker). Transfection was done using calcium phosphate method as follows: equal amounts of retroviral transfer vector and pCL-Eco packaging vector were mixed at 20 µg/ml each in 250 mM $CaCl_2$ solution at room temperature, then equal amount of 2x HBS (280 mM NaCl, 50 mM HEPES, 1.42 mM Na_2HPO_4, pH 7.05) was added dropwise with continuous vortexing to generate a fine-particle precipitate. After a brief incubation (2-5 min) the transfection mix was added dropwise to the cells in freshly changed medium, followed by gentle mixing. The medium was replaced 16 hrs after transfection and viral supernatants harvested 48 and 72 hrs post-transfection, filtered through sterile 0.45 µm filter and used for transduction on the same day. Femurs were obtained from mice and crushed in ice-cold PBS. The marrow cells were strained through a 70 µm cell strainer and lineage negative (Lin^-) cells were isolated using the Direct Lineage Cell Depletion Kit (Miltenyi Biotech, 130-110-470) according to the manufacturer's instructions. Following this, the cells were pre-stimulated in StemPro-34 SFM medium with 650 µL StemPro-34 supplement per 25 mL of media, 1x P/S, 1x GlutaMAX, 0.1 mM β-ME (Gibco), 50 µg/mL SCF, 10 µg/mL IL-3, 10 µg/mL IL-6 (Peprotech) for 48 hr at 37°C and 5% CO_2 prior to transduction. For retroviral transduction, a non-TC coated culture dishes were coated by with RetroNectin® (50 µg/ml in PBS, Clontech, T100B) overnight at 4°C, blocked with 2% BSA in PBS for 30 min at room temperature and loaded twice with viral supernatant by adding 4 ml per 35 mm well, centrifuging for 1 hr at 2000 xg, discarding and repeating with a second lot. The pre-stimulated Lin^- cells were added to the viral-coated plates and incubated at 37°C, 5% CO_2 for 48 hr. The transduced cells were transferred into progenitor outgrowth media (POM): RPMI-1640 (Invitrogen, 31870-074) supplemented with 10% ES-certified FBS (ThermoFisher Scientific, 16141079), 2 mM L-Glutamine, 0.1 mM β-ME, 100 IU/mL Pen-

Strep, 1 µM β-Estradiol (Sigma, E2758) and 5% filtered cell culture supernatant from a Flt3L-producing B16 melanoma cell line (a kind gift from E. Villablanca). Approximately 14 days after transduction, the HOXB8-FL lines grew homogenously and were cryopreserved while non-transduced bone marrow control cells had ceased to proliferate. Established HOXB8-FL cell cultures were subsequently grown at 37°C in a humidified 5% CO_2 incubator on non-TC treated plates at $0.2x10^6$-$2x10^6$ cells/mL and split every 48 hr. For expression of the AML1-ETO oncogene, established HOXB8-FL cell lines were transduced on two consecutive days with viral supernatant generated as described above using MSCV-AML1-ETO-IRES-EGFP ((Addgene plasmid # 12431). The proportion of transduced cells estimated by assessing GFP^+ fraction by flow cytometry at 24 hr after the second transduction was typically between 25-40%.

Screening for indels in HOXB8-FL cells

Screening for *Proser1* disruption was done using BstBI restriction fragment length polymorphism (RFLP) primers (FW: 5'-GAGACGTCACGACGGAATAGTA-3', RV: 5'-ATTCCCGTACCACAATGACTTC-3'), and Phire Hot Start II PCR Master Mix (Thermo Scientific, F125S) according to the manufacturer's instructions. The following PCR program was used: Denaturation: 98°C for 30 sec. PCR cycles (36X) of 98°C for 5 sec, 63.4^{o}C for 5 sec, 72°C 20 for sec, Final elongation: 72°C for 1 min. Hold: 4°C. 200 ng of the purified PCR product was subjected to digestion with 10U BstBI (NEB, R0519) for 1 hr at 65°C. The resulting fragments were analyzed by agarose gel electrophoresis, with an absence of a cut product representing a clonal culture with homozygous indels. To screen for *Tet3* disruption, we used T7 Endonuclease I (NEB, M0302S) assay per manufacturer's protocol. In brief, a fragment containing the target region was PCR-amplified from genomic DNA of individual clones using primers FW: 5'-CCACCTCTGAGCGCAGAGTG-3' and RV: 5'-GATGAACACAGTTCCTGACAG-3' and Q5 DNA polymerase (NEB, M0491S) with following PCR conditions: Denaturation: 98°C for 30 sec. PCR cycles (35X) of 98°C for 10 sec, 64°C for 30 sec, 72°C 20 for sec, Final elongation: 72°C for 2 min. Hold: 4°C. PCR products were then purified with PCR clean up kit (NEB, T1030S), annealed, incubated with T7EI and analyzed by agarose gel electrophoresis for the presence of cleavage which indicated a mutation. Putative mutant clones were further analyzed for the absence of PROSER1 and TET3 by Western Blotting. The target genomic regions of the western blot confirmed PROSER1 and TET3 KO clones were then PCR-amplified using Q5 DNA polymerase, cloned out using Strataclone Blunt PCR Cloning Kit (Agilent, 240207) and sequenced using M13R primer.

HOXB8-FL cell growth in semi-solid media

For growth assays under self-renewing conditions, four independent HOXB8-FL cell lines for each genotype were plated in triplicate at 500 cells/mL in semi-solid E2/Flt3 medium (MethoCult™ M3231 containing 2 mM L-glutamine and 100 IU/mL Penicillin-Streptomycin, with added 1 µM β-Estradiol (Sigma) and 5% B16-Flt3L supernatant). Colonies and cells were counted after 7 days of growth.

For serial replating assays in the context of AML1-ETO overexpression and in the absence of HOXB8 expression and Flt3L, four independent HOXB8-FL cell lines for each genotype were washed twice with PBS, re-suspended at 500 cells/mL in semi-solid medium as in [4] (MethoCult™ M3231 containing 2 mM L-glutamine and 100 IU/mL Penicillin-Streptomycin, with added 10 ng/mL IL-3, 10 ng/mL IL-6 and 50 ng/mL SCF (Peprotech, 213-13, 200-06 and 250-03), plated onto 35 mm dishes at 1 mL/dish, in duplicate, and incubated for 7 days. The subsequent rounds of re-platings at 7 day intervals were done in triplicate, with 7500 cells/mL in the second and 2000 cells/mL in the third, fourth and fifth rounds. Colonies and cells were counted and the contribution of GFP population assessed by flow cytometry after each round of re-plating.

Generation of FLAG-TET2 K562 and CAL-1 cells

K562 cells (ATCC, CLL-243) and CAL-1 cells (Kindly provided by T. Maeda) were cultured in RPMI-1640 supplemented with 1x L-Glutamine, 1x Pen/Strep, 10% FBS (heat inactivated) (Gibco) at 37°C, 5% CO_2. Cells were replated at 0.5-2x10^6 cells/mL every 48 hr. Full-length human TET2 (hTET2) with a N-terminal 2xFLAG tag was cloned into PiggyBAC-GW-Blast vector (PiggyBac-FLAG-hTET2-IRES-Blast, deposited at Addgene #85996) and transfected into K562 and CAL-1 cells with PiggyBac transposase (PBase) using Neon™ Transfection System 100 µl kit (Invitrogen). 2x10^6 cells were washed in PBS, re-suspended in 100 µL Buffer R, mixed with 0.0375 ng/µL PiggyBac transposase and 0.0375 ng/µL PiggyBAC-FLAG-hTET2-Blast plasmid, electroporated according to the manufacturer's instructions (K562: 1450 mV, 1 0ms, 3 pulses; CAL-1: 1750 mV, 20 ms, 1 pulse) and plated at 1x10^6 cells/mL in antibiotic-free medium. Transfected cells were cultured for 96 hr and then subjected to Blasticidin (Sigma) selection (K562: 32 mg/mL; CAL-1: 4 mg/mL) for 5 days at which point the untransfected control cells were no longer viable.

Immunoprecipitation

Cell lysates were prepared by 30 min incubation on ice in zwitterionic lysis buffer supplemented with protease inhibitors: 50 mM HEPES pH 7.5, 150 mM NaCl, 10 mM NaF, 0.5% C_7BzO (Sigma), 1X cOmplete protease inhibitors, EDTA free and 30min centrifugation at 20,000 x g. Concentrations were measured by Bradford assay and normalised. Immunoprecipitation was carried out on ≥1 mg protein in 1 mL pre-cleared lysate using 30 µL of a 50% (v/v) slurry of α-FLAG M2 affinity gel (Merck) and

incubation for 3 hr at 4°C with rotation. The beads were then washed 5X in ice-cold wash buffer supplemented with 1X protease inhibitors: 10 mM Tris-HCl pH 8, 1 mM EDTA, 150 mM NaCl, 1X cOmplete protease inhibitors, EDTA free (Roche, 11836170001). Proteins were eluted by resuspension in 2X LDS sample buffer (Invitrogen, NP0007) and incubation at 70°C with shaking at 1500 RPM for 15 min. Samples were supplemented with DTT to a final concentration of 0.1 M and boiled for 10 min before analysis by Western blotting.

Immunoprecipitation and mass spectrometry (IP-MS)

For analysis of eluted proteins by mass spectrometry, FLAG-IP was carried out as described above using 3mg protein in 1mL lysis buffer. After completing washes of the beads, 23ul of SDS lysis buffer (5% SDS, 100mM TEAB) and 20mM TCEP were added and incubated at 37°C. Immediately thereafter, freshly dissolved iodoacetamide was added to a final concentration of 20mM and incubated at 37°C for 10min. Then 2.5uL of 27.5% (v/v) phosphoric acid was added to the samples, followed by 165uL of S-trap binding buffer (100mM TEAB pH 8.5 in 90% aqueous methanol). Samples were loaded onto S-trap micro columns and the columns washed five times by addition of S-trap binding buffer and centrifugation (1min, 4000 x g). To digest the samples, 2.25 ug of MS-grade trypsin (Pierce, 90058) was resuspended in 50mM TEAB and loaded onto each column and incubated 2h at 47°C without shaking. The digested peptides were eluted by centrifugation with 100mM TEAB, followed by 0.2% aqueous formic acid and finally 50% aqueous acetonitrile. The three eluates for each sample were pooled, dried in a SpeedVac Vacuum concentrator, and sent for mass spectrometry analysis. Eluted peptides were analysed on an Orbitrap Exploris 480 Mass Spectrometer (Thermo Scientific) using a 120 min gradient elution and data-independent acquisition. The raw files were processed using Spectronaut® software [5] (Biognosys) and searched against Human Swiss-Prot including isoforms (February 2022). The parameters for the search included: Protein and Precursor Qvalue Cutoff (Experiment) were set to 0.01, with the Protein Qvalue Cutoff (Run) set to 0.05 as well as use of the 'Global imputation strategy' (imputed based on a random sampling from a distribution of low abundant signals taken across the entire experiment) to impute missing data in the IP samples. Finally, differential enrichment with multiple testing correction was determined using Perseus 1.6.14.0 [6] and plotted in Prism (GraphPad).

Western Blotting

Cell lysates were prepared as described in immunoprecipitation (K562 cells and CAL-1 cells) or lysed by suspension in 2X LDS sample buffer with 100 mM DTT and boiling for 10 min (HOXB8-FL cells). Between 5 and 50 µg protein was loaded, or loading was normalised to cell number (e.g. $2x10^5$ HOXB8-

FL cells per lane) and subjected to SDS-PAGE on a Bis-Tris or Tris-Acetate gel (ThermoFisher). Proteins were transferred to nitrocellulose membrane (Amersham) and probed using primary antibodies against FLAG (FLAG M2, Sigma F1804, 1:1000), human TET2 (D6B9Y, CST 18950, 1:1000), mouse TET2 (D6C7K, CST 36449, 1:1000) PROSER1 (PROSER1-N, In-house, 1:1000), OGT (Abcam ab96718, 1:1000), PSPC1 (Bethyl A303-206A, 1:1000), GAPDH (14C10, CST 2118, 1:10000). Imaging was carried out using photographic film or infrared fluorescence imaging (LI-COR Biosciences).

ChIP-seq

Biological replicate samples of wildtype, PROSER1 knockout, or TET2 knockout HOXB8-FL cell lines were crosslinked by resuspension in PBS with 1% formaldehyde (Thermo Scientific, 28906) at room temperature for 10 min. Quenching was performed by addition of glycine to a final concentration of 125 mM. Cells were washed with PBS and lysed by resuspension in SDS buffer: 50 mM Tris-HCl pH 8.1, 100 mM NaCl, 5 mM EDTA, 0.5% SDS supplemented with 1 mM Phenylmethylsulfonyl fluoride (PSMF). Chromatin was collected by centrifugation at 300 x g, 6 min, 20°C and resuspended in IP buffer: 50 mM Tris-HCl pH 8.6, 100 mM NaCl, 5 mM EDTA, 1.6% Triton X-100, 0.3% SDS. Sonication was carried out using a Bioruptor Pico (Diagenode) and fragmentation conditions (to obtain 100-500 bp fragments) were optimized for each experiment before continuing. Sonicated samples were cleared by centrifugation at 20,000 x g, 20 min, 4°C and the pellet discarded. Protein concentration was estimated by Bradford assay (BioRad, 500-0006) and the concentrations normalized across samples. SDS-free buffer: 50 mM Tris-HCl pH 8.1, 100 mM NaCl, 5 mM EDTA was used to dilute SDS in samples to a final concentration of 1% and the samples were precleared by 3 hr incubation with Protein G Sepharose beads (Cytiva, GE17-0618-01).

Immunoprecipitation was carried out overnight on 60 µg precleared chromatin in 1 mL of buffer. Antibody quantities were as follows: H3K4me1: CST 5326 (D1A9 Rabbit monoclonal, 10 µL to 60 µg chromatin), H3K27ac: ThermoFisher MA5-23516 (Mouse monoclonal, 4 µg antibody to 60 µg chromatin), H3K27me3: CST 9733 (C36B11 Rabbit monoclonal, 10uL to 60ug chromatin). Antibody-DNA complexes were immunoprecipitated by 3hr incubation with 30uL 50% slurry of Protein G Sepharose beads (Cytiva, GE17-0618-01) and washed three times with low salt wash buffer: 20 mM Tris-HCl pH 8.0, 150 mM NaCl, 2 mM EDTA, 0.1% SDS, 1% Triton X-100, twice with high salt wash buffer: 20 mM Tris-HCl pH 8.0, 500 mM NaCl, 2 mM EDTA, 0.1% SDS, 1% Triton X-100, and once with IP buffer with a final concentration of 0.1% SDS. Beads were then resuspended in decrosslinking buffer: 1% SDS and 100 mM $NaHCO_3$ and incubated at 65°C, shaking at 1200 RPM overnight. DNA was purified using the Monarch PCR & DNA cleanup kit. Libraries were prepared from 1-3 ng purified DNA

input using Truseq adapters (Illumina) and sequenced on a NovaSeq 6000 system with 150 bp paired-end sequencing.

ChIP-seq computational analysis

Paired-end Illumina reads obtained by sequencing were trimmed of adapter sequences and low-quality nucleotides using Trimmomatic, then mapped to the mouse genome (mm10) using Bowtie2 [7] with (--very-sensitive) preset settings. MarkDuplicates from Picard tools was used to de-duplicate mapped reads. Mapped reads were filtered to remove those from non-canonical chromosomal DNA and mtDNA. The normalized enrichment of H3K27me3 and H3K27ac ChIP-seq reads across unchanged and DNA hypermethylated CGIs in wildtype and PROSER1 knockout was visualised using EaSeq [8]. Peaks were called using MACS2 with paired-end settings (--format BAMPE --mfold 5 50 --bw 300) using a 0.05 p-adj threshold. In addition, H3K4me1 and H3K27me3 peaks were called as --broad, with a broad region cutoff of 0.1. Differential enriched peaks across replicates was defined using DiffBind [9]. Volcano plots of differential enriched peaks were plotted with Prism. Computational analysis was performed using the free public European Galaxy server usegalaxy.eu [10].

Enzymatic Methyl-seq (EM-seq)

Biological duplicate cell lines of wildtype, TET2 knockout and PROSER1 knockout HOXB8-FL cells were harvested and gDNA extracted using the Monarch Genomic DNA Purification Kit (New England Biolabs, T3010) to preserve genomic DNA integrity prior to sonication. Sonication was carried out using a bioruptor Pico (Diagenode) and fragmentation conditions (to obtain 300-450 bp fragments) were optimised for each experiment before continuing. EM-seq was performed using the NEBNext Enzymatic Methyl-seq Kit (New England Biolabs, E7120S) according to the protocol for use with Large Insert Libraries using 100 ng input DNA.

Enzymatic Methyl-seq (EM-seq) computational analysis

Raw EM-seq reads from the two biological replicate wildtype, TET2 KO, and PROSER1 KO HOXB8-FL samples were trimmed using TrimGalore and mapped against the mouse genome (mm10) using Bismark (https://github.com/FelixKrueger/Bismark) with standard setting allowing for 1000 bp insert size (--X 1000). Bismark deduplicate was used to remove PCR duplicates. Bismark methylation extractor was used with the following parameter setting (--paired-end --no-overlap --ignore_r2 3 --ignore_3prime_r2 1) to remove methylation call bias due to adaptor ligation and avoid scoring methylation calls twice in case of overlapping paired-end reads. Methylation calls in a CpG context were merged (combining CpG methylation calls from both strand of a single CG dinucleotide) to

produce a comprehensive "Methreport_CpGcomb_mm10" file containing summarised methylation calls for all CG sites in the mouse genome. All analysis was performed through the free public European Galaxy server usegalaxy.eu [10]

Individual methylation calls were imported into SeqMonk NGS visualisation and analysis tool (https://github.com/s-andrews/SeqMonk) using the QuasR import filter and input files with the following columns <chr><start><end><total reads><methylated reads>. Following a correlation analysis to determine reproducibility between biological replicates, the datasets were pooled to attain a collective coverage of ~18.9 million CpG sites, each supported by a minimum of 10 distinct EM-seq reads across all three genotypes – a condition required for all downstream analysis. Average hyper- or hypomethylation across different genomic regions was defined as >10% mean increase and >10% mean decrease, respectively. The resulting region sets were used for GREAT [3] gene ontology enrichment analysis using the 'Basal plus extension' setting. Datasets defining CGIs, gene bodies, non-CGI promoters (Promoters defined as -1500 bp to +500bp from a TSS, non-overlapping with a CGI) were obtained from UCSC table browser (GRCm38/mm10) and heterochromatic regions were obtained from ChromHMM annotation (https://github.com/guifengwei/ChromHMM_mESC_mm10). Active enhancers were defined as regions with peaks of H3K4me1 and H3K27ac (see ChIP-seq analysis described above) and non-overlapping with promoters or CGIs. Quantitation trend plots were generated using a 50 bp window size and 50 bp step size.

RNA-seq

Total RNA was isolated from biological replicate samples of FACS-sorted wildtype and PROSER1 knockout LSK cells using a RNeasy Plus Mini kit (Qiagen, 74134). RNA integrity was checked by gel electrophoresis on an Agilent Tapestation system. Libraries were prepared using the NEBNext Ultra II Directional RNA Library Prep kit (NEB, E7765) according to manufacturer's instructions.

RNA-seq computational analysis

To measure gene expression changes in protein coding genes, raw paired-end RNA-seq reads from the biological replicate wildtype and PROSER1 knockout LSK cells were trimmed using Trimmomatic and mapped to the mouse genome (mm10) using RNA-STAR [11] with paired-end settings. Gene annotation 'gencode.vM25.annotation.gtf.gz' was obtained from GENCODE release 25 (GRCm38.p6) and gene expression was summarized from mapped reads using FeatureCounts, with option (--countReadPairs) to enable counting of fragments from paired-end data. Differential expression was determined using DESeq2 with default parameters and plotted in Prism (GraphPad). Gene set enrichment analysis (GSEA) was conducted using GSEA software. The normalised expression data was filtered for lowly

expressed genes (using DESeq2 independent filtering) and used as input against a curated collection of hallmarks (‘h.all.v2024.1.Hs.symbols.gmt’) and chemical and genetic perturbations gene signatures (‘c2.cgp.v2024.1.Hs.symbols.gmt’) with weighted enrichment and Signal2Noise ranking parameters. The GSEA statistics (Normalized enrichment score (NES), Gene Ratio, and -$\log_{10}$(FDR)) from selected gene signatures associated with hematopoietic differentiation were plotted using ggplot2 (‘https://ggplot2.tidyverse.org’) and further modified in Illustrator (Adobe).

SUPPLEMENTARY REFERENCES

1. Gíslason MH, Demircan GS, Prachar M, et al. BloodSpot 3.0: a database of gene and protein expression data in normal and malignant haematopoiesis. *Nucleic Acids Res*. 2024;52(D1):D1138-D1142. doi:10.1093/nar/gkad993
2. Redecke V, Wu R, Zhou J, et al. Hematopoietic progenitor cell lines with myeloid and lymphoid potential. *Nat Methods*. 2013;10(8):795-803-. doi:10.1038/nmeth.2510
3. McLean CY, Bristor D, Hiller M, et al. GREAT improves functional interpretation of cis-regulatory regions. *Nat Biotechnol*. 2010;28(5):495-501. doi:10.1038/nbt.1630
4. Rasmussen KD, Jia G, Johansen J V, et al. Loss of TET2 in hematopoietic cells leads to DNA hypermethylation of active enhancers and induction of leukemogenesis. *Genes Dev*. 2015;29(9):910-922. doi:10.1101/gad.260174.115
5. Bruderer R, Bernhardt OM, Gandhi T, et al. Extending the limits of quantitative proteome profiling with data-independent acquisition and application to acetaminophen-treated three-dimensional liver microtissues. *Mol Cell Proteomics*. 2015;14(5):1400-1410. doi:10.1074/mcp.M114.044305
6. Tyanova S, Temu T, Sinitcyn P, et al. The Perseus computational platform for comprehensive analysis of (prote)omics data. *Nat Methods*. 2016;13(9):731-740. doi:10.1038/nmeth.3901
7. Langmead B, Salzberg SL. Fast gapped-read alignment with Bowtie 2. *Nat Methods*. 2012;9(4):357-359. doi:10.1038/nmeth.1923
8. Lerdrup M, Johansen JV, Agrawal-Singh S, Hansen K. An interactive environment for agile analysis and visualization of ChIP-sequencing data. *Nat Struct & Mol Biol*. 2016;23(4):349-357. doi:10.1038/nsmb.3180
9. Stark R BG. Bioconductor - DiffBind.
10. Community TG. The Galaxy platform for accessible , reproducible , and collabor ativ e data analyses : 2024 update. 2024;(May).
11. Dobin A, Davis CA, Schlesinger F, et al. STAR: Ultrafast universal RNA-seq aligner. *Bioinformatics*. 2013;29(1):15-21. doi:10.1093/bioinformatics/bts635
